# Environmental contamination across multiple hospital departments with multidrug-resistant bacteria pose an elevated risk of healthcare-associated infections in Kenyan hospitals

**DOI:** 10.1186/s13756-023-01227-x

**Published:** 2023-03-29

**Authors:** Erick Odoyo, Daniel Matano, Fredrick Tiria, Martin Georges, Cecilia Kyanya, Samuel Wahome, Winnie Mutai, Lillian Musila

**Affiliations:** 1 United States Army Medical Research Directorate-Africa, P.O. Box 606-00621, Nairobi, Kenya; 2 Kenya Medical Research Institute, P.O. Box 54840-00200, Nairobi, Kenya; 3 Independent researcher, Nairobi, Kenya; 4 Department of Medical Microbiology, Faculty of Health Sciences, University of Nairobi, P.O. Box 30197- 00100, Nairobi, Kenya

**Keywords:** Healthcare-associated infections, Hospital environment, Antibiotic resistance, Multi-drug resistance

## Abstract

**Background:**

Healthcare-associated infections (HAIs) are often caused by multidrug-resistant (MDR) bacteria contaminating hospital environments which can cause outbreaks as well as sporadic transmission.

**Methods:**

This study systematically sampled and utilized standard bacteriological culture methods to determine the numbers and types of MDR *Enterococcus faecalis*/*faecium, Staphylococcus aureus, Klebsiella pneumoniae, Acinetobacter baumannii*, *Pseudomonas aeruginosa, Enterobacter* species, and *Escherichia coli* (ESKAPEE) from high-touch environments of five Kenyan hospitals; level 6 and 5 hospitals (A, B, and C), and level 4 hospitals (D and E), in 2018. Six hundred and seventeen high-touch surfaces across six hospital departments; surgical, general, maternity, newborn, outpatient and pediatric were sampled.

**Results:**

78/617 (12.6%) of the sampled high-touch surfaces were contaminated with MDR ESKAPEE; *A. baumannii*, 23/617 (3.7%), *K. pneumoniae*, 22/617 (3.6%), *Enterobacter* species, 19/617 (3.1%), methicillin resistant *S. aureus* (MRSA), 5/617 (0.8%), *E. coli*, 5/617 (0.8%), *P. aeruginosa*, 2/617 (0.3%), and *E. faecalis* and *faecium*, 2/617 (0.3%). Items found in patient areas, such as beddings, newborn incubators, baby cots, and sinks were the most frequently contaminated. Level 6 and 5 hospitals, B, 21/122 (17.2%), A, 21/122 (17.2%), and C, 18/136 (13.2%), were more frequently contaminated with MDR ESKAPEE than level 4 hospitals; D, 6/101 (5.9%), and E, 8/131 (6.1%). All the sampled hospital departments were contaminated with MDR ESKAPEE, with high levels observed in newborn, surgical and maternity. All the *A. baumannii*, *Enterobacter* species, and *K. pneumoniae* isolates were non-susceptible to piperacillin, ceftriaxone and cefepime. 22/23 (95.6%) of the *A. baumannii* isolates were non-susceptible to meropenem. In addition, 5 *K. pneumoniae* isolates were resistant to all the antibiotics tested except for colistin.

**Conclusion:**

The presence of MDR ESKAPEE across all the hospitals demonstrated gaps in infection prevention practices (IPCs) that should be addressed. Non-susceptibility to last-line antibiotics such as meropenem threatens the ability to treat infections.

## Background

Healthcare-associated infections (HAIs) are among the leading threats to patient safety. Hospital patients are often predisposed to infections because of exposure to invasive devices during surgical procedures and possibly impaired or underdeveloped immunity [1]. In Kenya, the prevalence of HAIs is estimated to be 4.4 per 100 patient admissions, with the highest rates observed in medical, 5.1%, and pediatric, 4.9%, departments [2]. High rates of surgical site infections of up to 9.3% have been reported [3].

*Enterococcus faecalis*/*faecium, Staphylococcus aureus, Klebsiella pneumoniae, Acinetobacter baumannii*, *Pseudomonas aeruginosa, Enterobacter* species, and *Escherichia coli* (ESKAPEE) pathogens are the leading causes of HAIs globally and in Kenya [4, 5]. Consequently, the World Health Organization lists antibiotic-resistant ESKAPEE pathogens as high to critical priority pathogens for research and development of new antibiotics [6]. Infections caused by multi-drug resistant (MDR) ESKAPEE pathogens are of particular concern as they are associated with increased mortality and treatment costs [7, 8].

HAIs are frequently caused by bacterial pathogens that contaminate hospital environments[9]. These bacteria persist in hospital environments through the formation of biofilms and can withstand desiccation and resist disinfection [9, 10]. HAIs arising from contamination of frequently handled hospital surfaces or equipment regarded as high-touch surfaces[11], such as sinks, patients’ beds, and linens by bacterial pathogens, including; *Acinetobacter baumannii*, *Staphylococcus aureus*, carbapenem-resistant Enterobacterales, and vancomycin-resistant *Enterococcus* species, have been reported[12–14]. Microbial monitoring of these high-touch hospital environments can help determine the presence of contaminating pathogens and thus aid in implementing targeted infection prevention practices (IPCs) that may reduce HAIs. Our previous study determined the overall bacteria levels in Kenyan hospital environments and identified modifiable risk factors for improved infection control [15]. This study systematically sampled and characterized MDR ESKAPEE pathogens contaminating high-touch environments in five Kenyan hospitals and identified the highest-risk departments to target to reduce the risk of HAIs to patients in these hospitals.

## Materials and methods

### Study design

This descriptive laboratory-based study was conducted in five hospitals in Kenya, as previously described [15]. Briefly, one level six hospital (B), two level five hospitals (A and C), and two level four hospitals (D and E) were sampled. Hospital B is a 450-bed capacity national referral and teaching hospital. Hospitals A and C, with 168 and 270-bed capacities, respectively, are county referral hospitals, while Hospitals D and E, with 158 and 54-bed capacities, respectively, are level four facilities. Three departments identified by the hospital administration as having high levels of HAIs were selected for sampling in each hospital. In addition, the outpatient departments of the five hospitals were sampled. In total, five outpatient departments (hospitals A, B, C, D, and E), five pediatric departments (hospitals A, B, C, D and E), four surgical departments (hospitals B and E, and two surgical departments in hospital A), three maternity departments (hospitals C, D and E), three newborn departments (hospitals A, B, and D) and two general departments (hospitals D and E) were sampled.

### Sampling strategy

Sampling was carried out twice in each of the hospitals, between February and September.

2018. Swabs in neutralizing buffer (NB) (Puritan ESK sampling kit, Guilford, ME, USA) were used to sample 617 selected high-touch areas. High-touch areas, as classified in the guidelines for environmental infection control in healthcare facilities [16], are surfaces or equipment frequently handled by patients and clinicians, thus carrying a high risk for transmission of HAIs. For each surface, one swab was used. The surfaces sampled included items and areas close to the patient, such as; intravenous pole steering handles, intravenous tubing, patient bedding, bed rails, newborn incubators, tray tabletops, baby cots, bedside tabletops, baby weighing scale, room light switch plates, room inner doorknobs and clinician gowns. Surfaces in the bathroom, such as sinks, handrails, and toilet flush handles, were also sampled. In addition, equipment, including computer keyboards and mice, blood pressure cuffs, clinician cell phones, stethoscopes, and thermometers, were sampled. A sterile square frame measuring 500cm^2^ was used to define the swabbed area, while for smaller objects or surfaces, the surface area was approximated, and the whole surface area was swabbed. Samples were collected from clinician uniforms by swabbing the abdominal region and sleeve cuffs of the uniform. The swabs were then shipped at 4℃ to the testing lab at the Kenya Medical Research Institute (KEMRI), Nairobi, where they were processed within 36 h.

### Isolation and detection of MDR ESKAPEE pathogens

The NB solution containing the swab was vortexed and 100 µl was inoculated on respective chromogenic agars (CHROMagar, Paris, France) for isolation and detection of ESKAPEE pathogens; CHROMagar ESBL for *Enterobacterales*, CHROMagar MRSA for methicillin resistant *Staphylococcus aureus* (MRSA), CHROMagar VRE for vancomycin-resistant *Enterococcus* species and CHROMagar Acinetobacter for *Acinetobacter baumannii*. The inoculated plates were incubated aerobically at 37 °C for 24 h. The target MDR ESKAPEE was identified by observing the typical growth characteristics on the respective chromogenic agar. Three colonies of the target organisms were sub-cultured on Mueller Hinton agar (HIMEDIA, Mumbai, India) and incubated for 24 h to obtain pure bacterial isolates. Gram stain was performed for each isolate. Bacterial identification and antimicrobial susceptibility testing were performed on the VITEK 2 system per Clinical and Laboratory Standards Institute (CLSI) 2017 guidelines. AST-XN05 and AST-P580 cards were used for gram-negative and gram-positive antimicrobial susceptibility testing, respectively. *Staphylococcus aureus* 25,923 and *Escherichia coli* 25,922 were used for quality control. Bacterial isolates were categorized as MDR if they were non-susceptible to at least one antimicrobial drug in three or more therapeutically relevant antibacterial classes [17].

### Statistical analysis

Data were captured in excel sheets. All statistical analyses were performed on STATA (StataCorp. 2013. College Station, TX, USA). Descriptive statistics were expressed as percentages. Chi-square Fisher’s exact test was used to determine associations between the numbers and type of contaminating MDR ESKAPEE and the study hospitals and their departments. A *P*-value of ≤ 0.05 was considered statistically significant. The hospitals were classified as either higher level; level 6 hospitals (B), level 5 hospitals (A and C), or lower level; level 4 hospitals (D and E).

## Results

### Recovery of MDR ESKAPEE from the high-touch surfaces

A total of 617 hospital high-touch surfaces were sampled across the five study hospitals. Six hospital departments were sampled, including; surgical, general, maternity, newborn, outpatient and pediatric. 78/617 (12.6%) of the sampled high-touch surfaces across the study hospitals were contaminated with MDR ESKAPEE. The isolated MDR ESKAPEE pathogens included; *A. baumannii*, 23/617 (3.7%), *K. pneumoniae*, 22/617 (3.6%), *Enterobacter* species, 19/617 (3.1%), MRSA, 5/617 (0.8%), *E. coli*, 5/617 (0.8%), *P. aeruginosa*, 2/617 (0.3%), and *Enterococcus faecalis* and *faecium*, 2/617 (0.3%), (Table 1). *A. baumannii*, *K. pneumoniae* and *Enterobacter* species contaminated the widest range of hospital surfaces. The most frequently contaminated items were those found in patient areas, including patient beddings, newborn incubators and baby cots, department sinks, door knobs, and tray table tops. MDR ESKAPEE pathogens were also isolated from equipment such as intravenous pole steering handles, light switch plates, and a dial pad for a surgical table. Bathroom surfaces, bathroom sinks and a saline bathtub were also contaminated with MDR ESKAPEE.

**Table 1:** Distribution of MDR ESKAPEE pathogens on high-touch surfaces

MDR ESKAPEE	No. of isolated MDR ESKAPEE/No. of surfaces sampled (%)	High-touch surfaces contaminated with the MDR ESKAPEE pathogens
*Acinetobacter baumannii*	23/617 (3.7%)	*Patient areas*; bed rails, nursing chair, department sinks, patient beddings, door knob, tray table top *Equipment*; intravenous poles steering handle, suction tube *Bathroom areas*; saline bathtub
*Klebsiella pneumoniae*	22/617 (3.6%)	*Patient areas*; bed rails, patient beddings, newborn incubators, department sinks, baby cots, patient chair *Equipment*; an oxygen concentrator dial pad, room light switch plate *Bathroom areas*; bathroom sink
*Enterobacter* species	19/617 (3.1%)	*Patient areas*; bed rails, baby cots, newborn incubators, surgical table, patient beddings, inner door knobs, department sinks *Equipment*; dial pad for a surgical table
MRSA	5/617 (0.8%)	*Patient areas*; patient beddings, tray table top, bed rail
*Escherichia coli*	5/617 (0.8%)	*Patient areas*; bed rails, cupboard handle, newborn incubator *Equipment*; light switch plate
*Pseudomonas aeruginosa*	2/617 (0.3%)	*Patient areas*; newborn incubator *Bathroom areas*; bathroom sink
*Enterococcus faecalis* and *Enterococcus faecium*	2/617 (0.3%)	*Patient area*; newborn incubator
**Total**	**78/617 (12.6%)**	

### Distribution of MDR ESKAPEE pathogens across the different hospital levels

Higher-level hospitals were more frequently contaminated with MDR ESKAPEE than lower-level hospitals; level 6 hospital B, 21/122 (17.2%), level 5 hospitals A and C, 21/122 (17.2%), and 18/136 (13.2%), respectively, and level 4 hospitals, D, 6/101 (5.9%), and E, 8/131 (6.1%),. There was no significant association between the rates of contamination of each of the leading contaminants, MDR *A. baumannii*, MDR *K. pneumoniae* and MDR *Enterobacter* species, with the level of the hospital facility, *P* = 0.097, *P* = 0.721 and *P* = 0.729, respectively.

### Distribution of MDR ESKAPEE pathogens across the different hospital departments

Newborn and surgical departments were the most frequently contaminated with MDR ESKAPEE, 18/70 (25.0%) and 21/110 (19.1%), respectively (Table 2). *A. baumannii*, *K. pneumoniae*, and *Enterobacter* species accounted for 30/37 (81.1%) of MDR ESKAPEE contamination in these two departments. Conversely, the outpatient departments, 8/174 (4.6%), and general departments, 3/64 (4.7%), were the least frequently contaminated with MDR ESKAPEE pathogens.

**Table 2:** Distribution of MDR ESKAPEE pathogens across the hospital departments

MDR ESKAPEE	Newborn (*n* = 3)	Surgical (*n* = 3)	Maternity (*n* = 3)	Pediatric (*n* = 5)	General (*n* = 2)	Outpatient (*n* = 5)	Total
*Acinetobacter baumannii*	2	9	4	6	1	1	23
*Klebsiella pneumoniae*	8	6	5	1	0	2	22
*Enterobacter* species	4	3	5	2	2	3	19
*Escherichia coli*	1	2	0	2	0	0	5
*Pseudomonas aeruginosa*	1	0	0	0	0	1	2
MRSA	0	1	0	3	0	1	5
*Enterococcus faecalis*	1	0	0	0	0	0	1
*Enterococcus faecium*	1	0	0	0	0	0	1
**Total**	**18/70 (25.0%)**	**21/110 (19.1%)**	**14/80 (17.5%)**	**14/119 (11.8%)**	**3/64 (4.7%)**	**8/174** **(4.6%)**	**78/617 (12.6%)**

### Antibiotic susceptibility profiles of the isolated MDR ESKAPEE isolates

All the 23 *A. baumannii* isolates were non-susceptible to piperacillin, 23/23 (100.0%), cefepime, 23/23 (100.0%), and ceftriaxone, 23/23 (100%) (Table 3). 22/23 (95.6%) of the *A. baumannii* isolates were non-susceptible to meropenem. All the 22/22 (100.0%) *K. pneumoniae* isolates were non-susceptible to; piperacillin, cefepime, cefuroxime, cefuroxime-axetil, ceftriaxone, and aztreonam. Five of the twenty-two (22.7%) *K. pneumoniae* isolates were resistant to all the antibiotics tested except colistin. In addition, one of the twenty-three (4.4%) *A. baumannii* isolates was also resistant to all antibiotics tested except colistin. All the 19/19 (100.0%) *Enterobacter* species were non-susceptible to piperacillin, cefepime, cefuroxime, cefuroxime-axetil, ceftriaxone, and aztreonam. 3/5 (60%) of the isolated *E. coli* were non-susceptible to antibiotics piperacillin, cefepime, cefuroxime, cefuroxime-axetil, ceftriaxone, aztreonam, minocycline, tetracycline and sulfamethoxazole. All five *E. coli* isolates were susceptible to meropenem and tigecycline. Both the *P. aeruginosa* isolates were non-susceptible to ticarcillin-clavulanic acid and meropenem. At least 3/5 (60%) of the isolated MRSA were non-susceptible to cefoxitin, benzylpenicillin, oxacillin, erythromycin, clindamycin, gentamycin, levofloxacin, moxifloxacin, tetracycline and trimethoprim-sulfamethoxazole. Both the *E. faecium* and *faecalis* isolates were non-susceptible to erythromycin, levofloxacin, tetracycline and nitrofurantoin (Table 4).

**Table 3:** Antibiotic susceptibility profiles of the Gram-negative MDR ESKAPEE bacterial pathogens

Antimicrobial agent	*Acinetobacter baumannii*, n = 23 (%)		*Klebsiella pneumoniae*, n = 22 (%)		*Enterobacter cloacae*, n = 19 (%)		*Escherichia coli*, n = 5, (%)		*Pseudomonas aeruginosa*, n = 2 (%)	
	NS	S	NS	S	NS	S	NS	S	NS	S
Piperacillin	23 (100)	0	22 (100)	0	18 (94.7)	1 (5.3)	4 (80.0)	1 (20.0)	1 (50)	1 (50)
Ticarcillin-Clavulanic Acid	23 (100)	0	18 (81.8)	4 (18.2)	18 (94.7)	1 (5.2)	5 (100)	0	2 (100)	0
Cefuroxime	nd	nd	22 (100)	0	19 (100)	0	4 (80.0)	1 (20.0)	nd	nd
Cefixime	nd	nd	22 (100)	0	19 (100)	0	4 (80.0)	1 (20.0)	nd	nd
Ceftriaxone	23 (100)	0	22 (100)	0	19 (100)	0	4 (80.0)	1 (20.0)	nd	nd
Cefepime	23 (100)	0	22 (100)	0	19 (100)	0	3 (60.0)	2 (40.0)	nd	nd
Aztreonam	nd	nd	22 (100)	0	19 (100)	0	3 (60.0)	2 (40.0)	nd	nd
Meropenem	23 (100)	0	5 (22.7)	17 (77.3)	0	19 (100)	0	5 (100)	2 (100)	0
Moxifloxacin	nd	nd	11 (50.0)	11 (50.0)	3 (15.8)	16 (84.2)	2 (40.0)	3 (60.0)	0	2 (100)
Levofloxacin	6 (26.1)	17 (73.9)	10 (45.5)	12 (54.5)	3 (15.8)	16 (84.2)	2 (40.0)	3 (60.0)	nd	nd
Tetracycline	12 (52.2)	11 (47.8)	14 (63.6)	8 (36.4)	16 (84.2)	3 (15.8)	3 (60.0)	2 (40.0)	2 (100)	0
Minocycline	1 (4.3)	22 (95.7)	14 (63.6)	8 (36.4)	15 (78.9)	4 (21.1)	4 (80.0)	1 (20.0)	0	2 (100)
Tigecycline	2 (8.7)	21 (91.3)	nd	nd	0	19 (100)	0	5 (100)	2 (100)	0
Chloramphenicol	nd	nd	13 (59.1)	9 (40.9)	13 (68.4)	6 (31.6)	2 (40.0)	3 (60.0)	nd	nd
Colistin	0	23 (100)	0	22 (100)	1/ (5.3)	18 (94.7)	0	5 (100)	0	2 (100)
Trimethoprim	nd	nd	21 (95.5)	1 (0.5)	15 (78.9)	4 (21.1)	5 (100)	0	nd	nd

**Abbreviations**: NS, non-susceptible (Resistant or Intermediate); S, Susceptible; nd, antimicrobial sensitivity testing not done

**Table 4:** Antibiotic susceptibility profiles of the Gram-positive MDR ESKAPEE bacterial pathogens

Antimicrobial agent	MRSA, n = 5 (%)		*E. faecium* and *E. faecalis*, n = 2 (%)	
	NS	S	NS	S
Cefoxitin	5 (100)	0	nd	nd
Penicillin	5 (100)	0	nd	nd
Oxacillin	5 (100)	0	nd	nd
Erythromycin	5 (100)	0	2 (100)	0
Gentamycin	5 (100)	0	nd	nd
Clindamycin	5 (100)	0	nd	nd
Linezolid	0	5/5 (100)	0	2 (100)
Levofloxacin	4 (80)	1/5 (20.0)	2 (100)	0
Moxifloxacin	4 (80)	1/5 (20.0)	ND	nd
Tetracycline	3 (60)	2/5 (40.0)	2 (100)	0
Tigecycline	0	5/5 (100)	0	2 (100)
Nitrofurantoin	0	5/5 (100)	2 (100)	0
Trimethoprim-sulfamethoxazole	5 (100)	0	nd	nd
Teicoplanin	0	5/5 (100)	0	2 (100)
Vancomycin	0	5/5 (100)	0	2 (100)
Fusidic Acid	0	5/5 (100)	nd	nd

Abbreviations: NS, non-susceptible (Resistant or Intermediate); S, Susceptible; nd, antimicrobial sensitivity testing not done; MRSA Methicillin resistant S. *aureus*

## Discussion

This study sampled high-touch surfaces in five Kenyan hospitals and found that more than 12.0% were contaminated with MDR ESKAPEE pathogens. There was a risk of HAIs by MDR ESKAPEE pathogens in all the hospital departments sampled. In particular, items found in patient areas, such as newborn incubators and patient beddings, were frequently contaminated, posing a high risk of HAIs. Four departments, newborn, surgical, maternity, and pediatrics, had particularly worrying contamination rates of at least 10%, with the surgical and newborn having the highest rates of 25.0% and 19.1%, respectively.

Surgical site infections comprise the largest proportion of HAIs in developing countries, with an estimated incidence of 5.6 per 100 surgical procedures [18]. Caesarian sections are the most common surgical procedure in Kenya[19]. Longer durations of labor have been associated with a higher incidence of surgical site infections following caesarian-section in Kenyan hospitals [20], suggesting a role of the hospital environments in the acquisition of HAIs. The high contamination rate of the surgical and maternity departments by MDR ESKAPEE in this study supports this hypothesis. Newborn populations are particularly vulnerable to HAIs because of their immature immunity [1] and the high rate of contamination of newborn departments by the MDR ESKAPEE in this study may explain, in part, the high prevalence of HAIs observed in Kenyan pediatric departments [2]. The higher levels of MDR ESKAPEE contamination in higher-level hospitals than in lower-level hospitals may be linked to increased antibiotics selection pressure resulting from extensive use, particularly in critical care units or specialized departments such as the surgical departments. Patients with severe infections, trauma and those referred from lower-level hospitals often seek medical care in higher-level hospitals. These patients often require surgical interventions and the administration of antibiotics for patient care.

MDR *A. baumannii* was the most frequently isolated ESKAPEE pathogen, 23/617 (3.7%) and was found across all departments. Its ability to form biofilms and withstand desiccation [21, 22] enables it to persist in the hospital environment for long periods. Furthermore, it can maintain virulence even after prolonged desiccation and starvation [23], which partly explains its ability to cause frequent and prolonged hospital outbreaks [21]. As a result, *A. baumannii* has been linked to a wide range of HAIs, including ventilator-associated pneumoniae, skin and soft tissue infections, urinary tract infections, secondary meningitis and bloodstream infections that often affect critically ill patients [24], all of which have been associated with high mortality rates [25]. Data on the burden of *A. baumannii* infections in Kenya is not readily available. The available data is from facility-based studies, which show that *A. baumannii* is an important cause of infections in Kenya [26, 27]. *A. baumannii* has also been implicated in outbreaks in a teaching hospital in Kenya [28]. The high frequency of *A. baumannii* isolation in this study suggests an impending threat of hospital acquired *A. baumannii* infections. Typically, *A. baumannii* is intrinsically resistant to commonly prescribed antibiotics, such as first and second-generation cephalosporins, chloramphenicol, and aminopenicillins [25]. Carbapenem antibiotics are the main treatment option for MDR *A. baumannii* infections [25], yet in this study, 22/23 isolates were carbapenem non-susceptible. The use of quaternary ammonium compounds-based disposable wipes by in-house staff and chlorine-based disinfection is recommended to reduce contamination of hospital surfaces by carbapenem-resistant *A. baumannii* [29]. Terminal cleaning and enhanced cleaning of the high-touch surfaces [30] can help reduce the risk of *A. baumannii* HAIs at the study hospitals. None of the contaminating *A. baumannii* isolates were resistant to colistin and 22/23 were susceptible to minocycline, leaving these agents as treatment options for carbapenem-resistant *A. baumannii* strains [31]. The high rate of carbapenem resistance in*A. baumannii*isolates reflects an overreliance on carbapenem, a last-line antibiotic, for treating*A. baumannii*, while disregarding first- and second-line options such as tetracyclines and fluoroquinolones at the study hospitals.

*K. pneumoniae* is a significant cause of opportunistic HAIs, including; pneumoniae, urinary tract infections, skin and soft tissue infections, septicemia and endocarditis [32]. It is the most common cause of HAIs, including hospital outbreaks, in Kenya and Africa [4, 33]. In this study, MDR *K. pnuemoniae* was the second most frequently isolated MDR ESKAPEE pathogen, 22/617 (3.6%). It was isolated from newborn, surgical and maternity departments from high-touch items in near-patient areas, bathroom areas, and hospital equipment, reflecting its ubiquitous nature [32] and ability to persist in the hospital environment by forming biofilms [10, 34]. Equally important, *K. pneumoniae* has been implicated in several outbreaks and HAIs in neonatal units [35–38]. In Kenya, multi-drug resistant *K. pneumoniae* has been implicated in an outbreak in a neonatal critical care unit of a referral hospital in which six of the thirteen patients succumbed, a 46% case fatality rate [39]. Multi-drug-resistant *K. pneumoniae* was also identified as the common cause of blood-borne infections in newborn units of another referral hospital in Kenya [40]. Indeed, these reports highlight multi-drug-resistant *K. pneumoniae* as an important cause of HAIs in Kenyan hospitals. Therefore, the high contamination rate of surfaces and equipment in newborn departments in this study is of grave concern. All the *K. pneumoniae* isolates were resistant to aztreonam and third and fourth-generation cephalosporins, ceftriaxone and cefepime, respectively, which infers the production of extended-spectrum beta-lactamases and/or AmpC β-lactamases in addition to other resistance mechanisms [41]. Additionally, 22.7% of the *K. pneumoniae* isolates were also resistant to meropenem which infers the production of carbapenemases [42][43]. Resistance to last-line antibiotics such as meropenem threatens the ability to treat *K. pneumoniae* HAIs. Enforcing effective terminal cleaning of newborn incubators and enhanced cleaning of high-touch areas can help reduce the risk of *K. pneumoniae* HAIs in the newborn departments of the study hospitals. Additionally, safe disposal of diapers, cleaning of soiled articles with water and appropriately diluted disinfectants and soap, adherence to hand hygiene procedures, and limited staff rotations have been successfully employed to contain an outbreak in a neonatal unit [44].

*Enterobacter* species are associated primarily with HAIs [45] and were the third most frequently isolated MDR ESKAPEE pathogen in this study, 19/617 (3.1%). It was isolated across all the departments. Third-generation cephalosporins are known to induce variants of AmpC beta-lactamases in *Enterobacter* species, resulting in resistance [46], and their widespread use in Kenyan hospitals [47, 48] could be providing a selective pressure that favors *Enterobacter* species and the emergence of cephalosporin resistance. Because of innate resistance to first and second-generation cephalosporins, treatment of *Enterobacter* infections is often limited to carbapenems, fluoroquinolones, and aminoglycosides [45]. Fourth-generation cephalosporins have been used to treat Enterobacter infections due to their relative stability to AmpC beta-lactamases in the absence of extended-spectrum beta-lactamases [45]. This study, however, found all the *Enterobacter* isolates resistant to cefepime, a fourth-generation cephalosporin, which further limits treatment options for *Enterobacter* infections in the study hospitals. There was, however, a low rate of resistance to fluoroquinolones, moxifloxacin, and levofloxacin, while meropenem was active against all the *Enterobacter* isolates from the study hospitals. Interestingly, one isolate was resistant to colistin while retaining susceptibility to other drug classes, which may be attributed to chromosomal mutations or acquired resistance genes. However, a more reliable colistin test will be necessary to rule out any false resistance often observed with colistin testing in automated platforms.

This study isolated two isolates of *P. aeruginosa* from high-touch surfaces. *P. aeruginosa* is a common contaminant in hospital environments, particularly moist surfaces such as sinks and taps [49, 50], which were sampled in this study. The media used to detect *P. aeruginosa* was not the optimal selective media which could have limited the ability to detect it from the hospital environment. Similar to *A. baumannii*, the two isolates of *P. aeruginosa* in this study were resistant to meropenem but retained susceptibility to the fluoroquinolone moxifloxacin further highlighting an overreliance on carbapenem for treatment at the study hospitals. Putting carbapenems on watch lists and limiting access without approval can help limit increasing levels of carbapenem resistance in the study hospitals.

At least 3/5 *E. coli* isolates were non-susceptible to third-generation cephalosporins, which may infer the production of extended-spectrum beta-lactamases. However, all five isolates of E. coli were susceptible to last-line antibiotics such as meropenem and tigecycline.

The Gram-positive MDR ESKAPEE pathogens, MRSA and *Enterococcus faecalis/faecium*, had high levels of resistance to first-line antibiotics, including erythromycin, levofloxacin, and tetracycline, while they were susceptible to last line antibiotics; vancomycin, teicoplanin, and tigecycline. This study isolated MRSA and *Enterococcus faecalis/faecium* mainly from the newborn and pediatric departments, which signifies a high risk of HAIs to these younger patient populations. CHROMagar VRE should ideally limit the growth of contaminating bacteria and vancomycin-susceptible *Enterococcus* species. Despite this, in this study, the detected isolates of the *Enterococcus faecalis/faecium* were susceptible to vancomycin, indicating that the media may be permissive to their growth and antimicrobial sensitivity testing is necessary to confirm whether the recovered isolates are indeed vancomycin-resistant. Gram-negative MDR ESKAPEE pathogens were isolated in greater numbers than Gram-positive MDR ESKAPEE, which may be linked to IPC practices such as using contaminated cleaning materials, which has been found to mainly replaceram-positive cocci with Gram-negative bacilli [51]. In a previous study conducted in the same hospitals [15], storing wet mops was predictive of increased bacterial loads, reflecting that this poor cleaning practice contributes to the spread of bacterial contamination.

### Study limitations

Molecular characterization of the isolates from this study would provide more information on the MDR ESKAPEE strain types found within these hospitals. Further, a comparison with clinical strains could confirm the transmission patterns and ascertain that these pathogens are causes of HAIs in the study hospitals. The low rate of detection of *Pseudomonas aeruginosa*, a common cause of HAIs [52, 53], may be due to the failure to use a suitable culture media for its detection in the study.

## Conclusion

The widespread presence of MDR ESKAPEE contamination in the study hospital environments suggests low compliance to IPC practices in Kenyan hospitals, which are already outstretched by challenges such as poor water supply, frequent electricity outages, sporadic supply of critical cleaning reagents and personnel, among others. There was a high risk of HAIs by MDR ESKAPEE pathogens across all the sampled hospitals. Non-susceptibility to last-line antibiotics such as meropenem threatens the ability to treat HAIs. The study hospitals could benefit from strengthening diagnostic capacity for antimicrobial sensitivity testing for judicious antibiotics prescription practices. In addition, enforcing terminal cleaning of patients’ beds and newborn incubators, hospital environment biomonitoring around high-touch areas, antibiotic stewardship programs, enforced hand hygiene, and adequate and frequent cleaning of high-touch areas, as previously described [15] can help reduce the risk of HAIs at the study hospitals.

## Data Availability

All data generated or analyzed during this study are included in this published article.

## List of abbreviations

HAIs: Healthcare-associated infections
MDR: Multidrug-resistant
ESKAPEE: *Enterococcus faecalis*/*faecium, Staphylococcus aureus, Klebsiella pneumoniae, Acinetobacter baumannii*, *Pseudomonas aeruginosa, Enterobacter* species, and *Escherichia coli*
IPCs: Infection prevention control practices
NB: Neutralizing buffer
KEMRI: Kenya Medical Research Institute
CLSI: Clinical and Laboratory Standards Institute
WRAIR: Walter Reed Army Institute of Research
R: Resistant
NS: Non-susceptible (Resistant or Intermediate)
S: Susceptible
nd: Antimicrobial sensitivity testing not done
